# Developing best practices for engaging and involving palliative care patients in research in sub-Saharan Africa

**DOI:** 10.1186/s40900-026-00987-1

**Published:** 2026-10-08

**Authors:** Kennedy Nkhoma, Eve Namisango, Emmanuel Luyirika, Patience Asiimwe, Denis Olodi, Paul Ebusu, Jane Bates, Duncan Kwaitana, Dorothee Van Breevoort, Modai Mnenula, Dickson Dick Chifamba, Lovemore Mupaza, Edwina Beryl Opare-Lokko, Adwoa Bemah Boamah Mensah, Catherine Evans, Richard Harding

**Affiliations:** 1 Florence Nightingale Faculty of Nursing Midwifery and Palliative Care, Cicely Saunders Institute, King’s College London, London, UK; 2 African Palliative Care Association, Kampala, Uganda; 3 Uganda Cancer Society, Kampala, Uganda; 4 Department of Family Medicine, School of Medicine and Oral Health, Kamuzu University of Health Sciences, Private Bag 360, Blantyre, Malawi; 5 Island Hospice and Healthcare, 6 Natal Road, Belgravia, Harare Zimbabwe; 6 Ghana College of Physicians and Surgeons, 54 Independence Avenue, Ridge, Accra Ghana; 7 Department of Nursing and Midwifery, College of Health Sciences, Kwame Nkrumah University of Science and Technology, Kumasi, Ghana; 8 Sussex Community NHS Foundation Trust, Brighton, Brighton and Hove UK

**Keywords:** Serious illness, Multimorbidity, Sub-Saharan Africa, Patient and public involvement and engagement

## Abstract

**Background:**

Patient and public involvement and engagement (PPIE) are pivotal to maximising the impact of research on clinical care. Despite growing recognition of its importance, there remains limited evidence on best practices for implementing meaningful and contextually appropriate PPIE particularly in sub-Saharan Africa.

**Aim:**

This study aimed to explore patients and caregivers perspectives on meaningful engagement and involvement in palliative care research in Africa and identify contextually appropriate recommendations for establishing, supporting and sustaining PPIE structures and networks.

**Methods:**

This multi-national, qualitative cross-sectional study was conducted across four sub-Saharan African countries. We conducted qualitative studies across four African countries. In Ghana, Malawi, and Zimbabwe, in-depth interviews were conducted with older adults receiving primary or palliative care for chronic conditions. In Uganda, two focus group discussions (FGDs) were conducted with adult cancer patients. Topic guides explored: (1) the perceived added value of PPIE in research; (2) how patients and caregivers wished to engage in research; (3) potential roles within the research process; (4) preferred structures for PPIE activities; (5) capacity-building and training needs; and (6) support and resources required for effective participation. All interviews and FGDs were audio-recorded, transcribed verbatim, and analysed using framework analysis.

**Results:**

A total of 94 participants were recruited, comprising 57 patients and 37 caregivers (no caregivers were recruited in Uganda). Participants viewed PPIE as a platform for patient empowerment and meaningful engagement in research. Key areas in which participants wished to be involved included identifying research priorities, participating in ethical review processes, supporting patient identification and recruitment, contributing to data collection, engaging in research dissemination, and prioritising recommendations for patient care and health policy. Preferred PPIE formats included workshops and panel discussions, with quarterly meetings suggested. Training needs focused on capacity building, empowerment for PPIE roles, and preparing materials for dissemination.

**Conclusion:**

This study provides evidence-based recommendations for developing and sustaining meaningful PPIE in palliative care research in sub-Saharan Africa. Our findings contribute to the formalisation of PPIE in African palliative care research. Further work is needed to implement, refine and evaluate these approaches across different settings.

**Clinical trial number:**

Not applicable.

## Abstract (fr)

**Why did we do this study?**

Patients and family caregivers have valuable knowledge and experiences that can improve health research. However, in sub-Saharan Africa, they are rarely involved in planning, conducting, or sharing research. We wanted to understand how people living with serious illnesses and their caregivers would like to be involved in research and what support they would need.

**What did we do?**

We recruited 94 patients and caregivers from Uganda, Ghana, Malawi, and Zimbabwe. Through interviews and group discussions, we asked about their experiences, how they would like to contribute to research, what might help or prevent their involvement, and support needed to effectively be involved.

**What did we find?**

Participants were enthusiastic about being involved in research. They wanted to help identify research priorities, review study materials, recruit participants, collect and interpret data, and share research findings with their communities. They also highlighted the need for training, accessible meeting venues, and financial support for transport and meals. Participants emphasised the need for researchers to respect local languages, cultures, and community structures.

**Why is this important?**

Our findings provide participants informed recommendations for developing meaningful and sustainable patient and public involvement and engagement in palliative care research in sub-Saharan Africa. The findings can help researchers and institutions to develop contextually appropriate approaches that recognise patients and caregivers as active partners in research. This study recommends for further research to implement, refine and evaluate these approaches across different settings.

## Background

By 2050, approximately 80% of the world’s older population will be living in low- and middle-income countries (LMICs) [1]. World Health Organization (WHO) mortality projections indicate that by 2060, an estimated 39 million deaths in LMICs will be associated with serious health-related suffering, accounting for 83% of all global deaths. This represents a 153% increase within low-income countries between 2016 and 2060 [2]. These trends are driven primarily by diseases of ageing, including cancer, cerebrovascular disease, chronic lung disease, and dementia.

The growing burden of non-communicable diseases (NCDs) in LMICs has been recognised for over a decade. In 2008, the WHO reported that 36 million of the 57 million global deaths were due to NCDs principally cardiovascular disease, cancer, chronic respiratory disease, and diabetes with nearly 80% occurring in developing countries and approximately 9 million deaths occurring before the age of 60. In response, the 2011 United Nations Political Declaration on NCDs highlighted the urgent need for strengthened global, regional, and national action to prevent and control NCDs, in line with the right to the highest attainable standard of physical and mental health.

Despite this growing need, access to palliative care remains profoundly inequitable. The WHO estimates that only 10% of the 20 million people who require palliative care each year receive it, with the majority of provision concentrated in high-income countries, despite 80% of need being in LMICs [3]. Progressive illness in LMICs places a substantial burden on patients and their families particularly women, who provide most informal care with healthcare costs in the last year of life accounting for up to 20% of household expenditure [4–6].

The increasing prevalence of life-limiting conditions, together with uncertain prognoses and high symptom burden among people with cancer and non-cancer chronic conditions, highlights the need for research that responds to patients’ and caregivers’ priorities. Patient and public involvement and engagement (PPIE) can help shape research activities to improve the relevance and acceptability of healthcare research and its potential contribution to care delivery. Public involvement in research refers to research conducted *with* or *by* members of the public, rather than *to*, *about*, or *for* them, while public engagement encompasses activities that share and communicate research knowledge with the wider public [7]. PPIE represents a partnership approach that helps ensure research remains relevant, acceptable, and responsive to the needs and priorities of affected populations and is increasingly recognised as a prerequisite for high-quality research.

Meaningful involvement of patients and caregivers enables the development of medical, technological, and service innovations that are aligned with real-world needs and priorities. Research has reported research-based approaches to PPIE development in high income countries [8]. Evidence from settings with established PPIE infrastructures demonstrates that involving stakeholders can improve the relevance of research questions, enhance participant recruitment, and strengthen dissemination and policy uptake [9, 10]. However, approaches developed in high-income countries may require adaptation to different cultural, social and health-system contexts. A systematic review of PPIE in LMIC health research highlighted a paucity of literature describing effective approaches and their impact [11]. In sub-Saharan Africa, particularly within palliative care research, involving patients with cancer and non-cancer chronic conditions, there remains a need for evidence on how meaningful PPIE can be established and sustained in contextually appropriate ways. Addressing this gap is critical to ensuring that research reflects the priorities of patients, caregivers and communities and contributes to equitable, patient-centred research and care delivery.

This work was informed by the McCormack and McCance Person-centred Practice Framework, which conceptualises person-centred care as a collaborative partnership between healthcare professionals, patients, families, and communities [12]. Although originally developed to guide healthcare delivery, its principles are equally relevant to patient and public involvement and engagement (PPIE), recognising patients and caregivers as active partners in shaping research rather than passive participants.

We aimed to explore patients and caregivers’ perspectives on meaningful engagement and involvement in palliative care research in Africa and identify contextually appropriate recommendations for establishing, supporting and sustaining PPIE structures and networks.

## Methods

### Study design

This was a multi-national, qualitative cross-sectional study conducted across four sub-Saharan African countries between November 2020 and January 2022.

### Study setting and participants

In Uganda, participants were recruited from the Uganda Cancer Institute and Kiruddu National Referral Hospital. In Ghana, participants were recruited from Korle Bu Teaching Hospital and Tetteh Quarshie Memorial Hospital (T.Q.M.H.). In Malawi, recruitment took place at Mangochi District Hospital and Luwenya Health Centre. In Zimbabwe, participants were recruited from Mahusekwa and Mufakose Hospitals.

### Eligibility criteria

Patient inclusion criteria were as follows. In Uganda, adult cancer patients (aged 18 years or older) In Ghana, Malawi, and Zimbabwe inclusion criteria were older adults in line with the WHO definition for research in Africa, i.e. aged ≥ 50 years [13], and living with at least two serious illnesses. Serious illness was defined as *“a condition that carries a high risk of mortality*,* negatively impacts quality of life and daily function*,* and/or is burdensome in terms of symptoms*,* treatments*,* or caregiver stress.”* Such conditions are not necessarily advanced but are characterised by clinical uncertainty and would benefit from greater intervention in terms of patient outcomes and health system costs [14]. These were defined as cancer, heart disease, chronic respiratory disease, stroke, and diabetes. In all settings, additional criteria were sufficient cognitive capacity to provide informed consent and participate in data collection, and willingness to be audio-recorded.

Patient exclusion criteria included being below the minimum eligible age, lacking sufficient proficiency in English or the relevant local language (Luganda, Ewe, Chichewa, or Shona) to participate, or being unable to provide informed consent.

Family caregivers were defined as “unpaid, informal providers of one or more physical, social, practical, and emotional tasks. In terms of their relationship to the patient, they may be a friend, partner, ex-partner, sibling, parent, or other blood or non-blood relative” [25]. Family caregivers were eligible if they were aged 18 years or older, able to provide informed consent and willing to be audio-recorded. Exclusion criteria for caregivers were aged 17 years or below, unwilling to be audio-recorded.

### Recruitment procedures

In Uganda, participants were identified by social workers at the Uganda Cancer Institute, with mobilisation led by the Uganda Cancer Society. In Ghana, Malawi, and Zimbabwe, participants were identified through attending clinicians in primary care settings, including clinic managers, medical doctors, and nurse managers. Information sheets were provided and read aloud to potential participants. Additional recruitment was supported through non-governmental organisations working with older adults, as well as community leaders involved in home-based and palliative care services. Interested participants were referred to local principal investigators (Eve Namisango (EN) in Uganda, Edwina Beryl Opare-Lokko (EBO) in Ghana, Jane Bates (JB) in Malawi, and Dickson Chifamba (DC) in Zimbabwe), who provided further study information and obtained written informed consent. Data collection dates and venues were agreed upon following consent.

### Data collection

In Uganda, two focus group discussions were conducted, stratified by gender (one male group and one female group) to facilitate open discussion and minimise the influence of gender power dynamics on participants’ responses, an approach recommended for qualitative research on potentially sensitive topics [15]. In Ghana, Malawi, and Zimbabwe, individual in-depth interviews were conducted. Semi-structured topic guides were used to explore participants’ perspectives on: preferred ways of engaging in research, potential roles across the research cycle (including priority setting, study design, recruitment, data collection, analysis, dissemination, and impact), training, learning, and mentorship needs, support mechanisms and appropriate reimbursement for participation.

All participants completed a demographic questionnaire capturing age, gender, participant role (patient, caregiver, or public representative), diagnosis, religion, place of residence, and years since diagnosis. All interviews and focus group discussions were audio-recorded and transcribed verbatim then pseudonymised for reporting.

### Data management and analysis

Audio recordings were securely stored on password-protected computers in each participating country. Sample characteristics were descriptively analysed using Stata version 15 [16]. Qualitative data were imported into NVivo version 12 for analysis.

Data were analysed using a framework approach [17], with all participant data analysed jointly from a single coding framework developed inductively from the data. This enabled comparison across participant groups and countries and to develop a comprehensive understanding of participants’ views for each theme. The initial coding framework was developed inductively by Kennedy Nkhoma (KN) using a subset of transcripts, including two patient and two caregiver interviews from three countries (Ghana, Malawi, and Zimbabwe). This preliminary framework was then applied by EN to one focus group discussion from Uganda and modified.

The coding framework was subsequently shared with researchers from Ghana (Adwoa Bemah Boamah Mensah (ABBM), Edwina Beryl Opare-Lokko (EBO)), Malawi (Duncan Kwaitana (DK), Dorothee Van Breevoort (DVB), Modai Mnenula (MM)), and Zimbabwe (Lovemore Mupaza (LM) and DC), who independently applied it to an additional sample of two patient and two caregiver transcripts each. Researchers were encouraged to inductively add new codes where necessary. The research team then convened a virtual meeting to review the coding framework, discuss discrepancies, and reach consensus. All differences were resolved through discussion.

KN and DVB subsequently merged all coding contributions into a single, refined framework, which was used to code all remaining transcripts by the team. KN reviewed all coded transcripts and liaised with country teams to clarify any emerging issues.

Comparative analysis within the framework enabled identification of both common themes across countries and context-specific differences. Main themes and subthemes were defined to ensure internal consistency across the dataset. Illustrative quotations were selected to support each theme and are presented alongside participant characteristics and country of origin to demonstrate representation across the breadth of the sample.

### Ethics approval

This study was reviewed and approved by the Uganda Cancer Institute Ethics Review Committee REF UCI REC REF-04-202 and UNCST. In Ghana the study was approved by Ghana Health Service Ethics Review Committee GHS/RDD/ERC-ADMIN/REN/APP/22, in Malawi by College of Medicine research ethics committee P.08/20/3108 and in Zimbabwe the Medical Research Council of Zimbabwe MRCZ/A/275 approved the study. This study also received ethical approval from the Health Faculties Research Ethics Sub committees at King’s College London (Reference Nos. HR-19/20-18524 and HR-19/20-17229).

## Results

### Sample characteristics

A total of 94 participants took part in the study, comprising 57 older adults/patients and 37 caregivers. In Uganda, 16 patients (8 men and 8 women) participated in focus group discussions lasting approximately 90 min. No caregivers were recruited in Uganda. In Ghana, 12 older adults and 10 caregivers participated; in Malawi, 15 older adults and 15 caregivers participated; and in Zimbabwe, 14 older adults and 12 caregivers participated.

Participant characteristics varied across study sites (Table 1). Overall, the mean age of patients was 58.9 years (SD 11.6), while caregivers had a mean age of 40.8 years (SD 3.8). Among patients, 61.4% were female, over half **(**54.4%**)** identified as Christians, and approximately one-third **(**33.9%**)** had completed secondary education. Nearly half **(**43.9%**)** were unemployed, with farming being the second most common occupation (26.3%). Participants represented a range of chronic illnesses, including hypertension, diabetes, stroke, heart failure, arthritis, HIV and cancer; all Ugandan participants were living with cancer. Among caregivers, most were female (78.4%), Christian (64.9%), had completed secondary education (51.4%), and were most commonly siblings (35.1%) or parents (27.1%) of the patient.

**Table 1:** Sociodemographic characteristics of study participants by participant group and country

Characteristic	Patients (Ghana, Malawi, Uganda & Zimbabwe) (*n* = 57)	Caregivers (Ghana, Malawi & Zimbabwe) (*n* = 37)
**Country, n (%)**		
Ghana	12 (21.0)	10 (27.1)
Malawi	15 (26.3)	15 (40.5)
Zimbabwe	14 (24.6)	12 (32.4)
Uganda	16 (28.1)	–
**Age**,** mean (SD)**,** years**	58.9 (11.6)	40.8 (3.8)
**Sex**,** n (%)**		
Female	35 (61.4)	29 (78.4)
Male	22 (38.6)	8 (21.6)
**Education** ^†^, **n (%)**		
No education	12 (21.4)	2 (5.4)
Primary school	14 (25.1)	9 (24.3)
Secondary school	19 (33.9)	19 (51.4)
Tertiary education	11 (19.6)	7 (18.9)
**Religion**,** n (%)**		
Christianity	31 (54.4)	24 (64.9)
Islam	15 (26.3)	9 (24.3)
Orthodox	11 (19.3)	4 (10.8)
**Occupation**,** n (%)**		
Not employed	25 (43.9)	10 (27.1)
Farmer	15 (26.3)	9 (24.3)
Business	8 (14.0)	3 (8.1)
Temporary employment	2 (3.5)	–
Full-time employment	5 (8.8)	7 (18.9)
Student	–	5 (13.5)
Casual labour	–	3 (8.1)
Retired	2 (3.5)	–
**Diagnosis**,** n (%)**		
Hypertension and diabetes	11 (19.3)	–
HIV and cancer	6 (10.5)	–
Hypertension, diabetes and cancer	4 (7.0)	–
Stroke, hypertension and diabetes	8 (14.0)	–
Heart failure and hypertension	4 (7.0)	–
Hypertension, diabetes and arthritis	5 (8.8)	–
Cancer, hypertension and arthritis	3 (5.3)	–
Cancer	16 (28.1)	–
**Relationship to patient**,** n (%)**	–	
Sibling	–	13 (35.1)
Parent	–	10 (27.1)
Partner/spouse	–	6 (16.2)
Son/daughter/niece/granddaughter	–	5 (13.5)
Grandparent	–	3 (8.1)

† One participant had missing education data

### Main findings

**Fig. 1:**
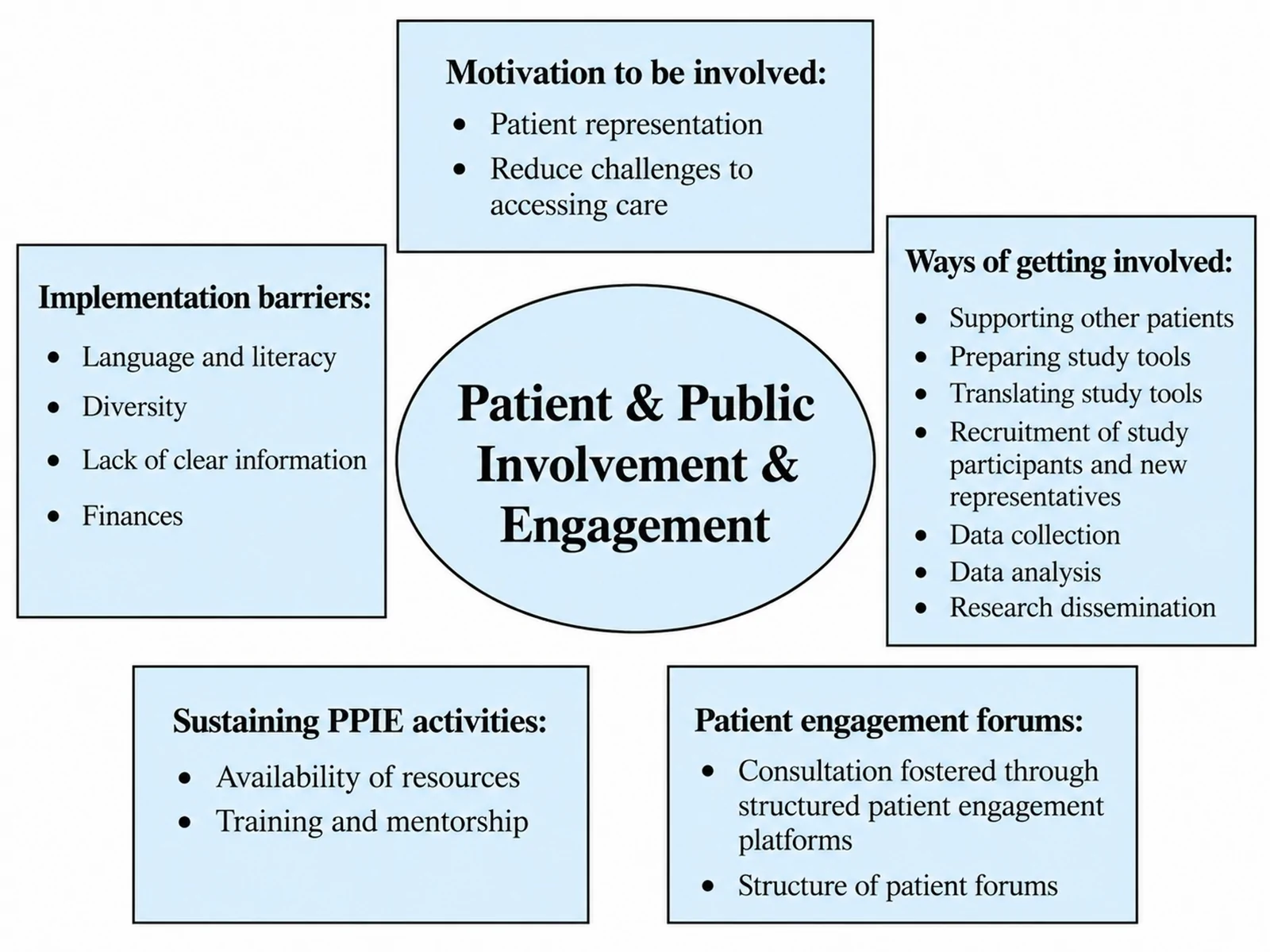
Summary of themes and subthemes

We identified five broad thematic areas (1) Motivation to get involved in palliative care research activities (2) Ways of getting involved in palliative care research, (3) Patient engagement forums, (4) Sustainability of patient and public involvement and engagement activities, and (5) Barriers to the implementation of PPIE activities. These are summarised in Fig. 1.

#### Motivation to get involved in palliative care research activities

##### Patient representation

Patient participants expressed a strong desire to be involved in research in order to represent themselves and other patients in similar situations. They emphasised that patient representation is essential for bridging the gap between healthcare workers, service providers, researchers, and patients. Participants noted that many patients are often reluctant to share their views openly due to fear of being identified or treated differently by healthcare providers.

Findings from the focus group discussions indicated that the formation of patient representative groups would facilitate more effective mobilisation for research participation and create safer spaces for patients to freely express their views and opinions without concerns about confidentiality breaches. Participants highlighted that such structures could provide leadership and enable patients to engage meaningfully in platforms such as research committees. Overall, there was a clear call for the establishment of patient groups with designated representatives to advocate on behalf of the wider patient community

Some patients feel used during studies so involving them will make them more willing to participate and give honest responses. Improves relationship between patients and researchers (Focus group discussion, Women’s group, Uganda).

We also need someone to represent us in pushing for information dissemination from research taken. (Focus group discussion, Men’s group, Uganda).

As cancer patients we are left behind many times. Sometimes we have challenges but have no opportunity or avenue to express our challenges (Cancer patient, Zimbabwe, P006).

##### Reduce challenges to access care

Participants expressed strong enthusiasm and willingness to engage in PPIE activities. Many described experiencing significant challenges in accessing care but reported having limited or no platforms through which to share these experiences. They believed that their lived experiences could provide valuable insights for the wider public and serve as catalysts for meaningful change within healthcare systems.

Consequently, participants viewed involvement in palliative care research as a worthwhile investment of their time and resources, with the potential to contribute to societal transformation and improvements in care delivery.

As cancer patients we are left behind many times. Sometimes we have challenges but have no opportunity or avenue to express our challenges (Cancer patient, Zimbabwe, P006).

One caregiver further explained that research provided a rare opportunity for patients and families to have their experiences heard and used to improve future care:

The goodness is that since my brother became ill, doctors have never come to ask him the progress of this illness like the way you have done. This is our luck for me and the patient to be asked these things. You will make use of the information and make decisions on what should be done.**(**Caregiver, Malawi, C013).

#### Ways of getting involved in research

Patients proposed to be involved in the following core activities; (a) patient support, (b) preparing study tools, (c) translating study tools, (d) recruitment of participants and patient representatives, (e) data collection, (f) data analysis, and (g) research dissemination.

##### Supporting other patients

Participants noted that PPIE provided valuable opportunities for engagement and had tangible benefits for some patients, including improved access to information, a heightened sense of being valued, and increased peace of mind. Participants further observed that, with appropriate training, mentorship, and empowerment, they could draw on their lived experience of serious illness to provide peer support and counselling to fellow patients.

Have patients participate in PPI because they would be more willing and open to share with fellow patients (Focus group discussion, Men’s group, Uganda).

##### Preparing study tools

Participants emphasised that clear articulation of what research entails is essential to enable patients to engage freely and participate meaningfully. They reported that researchers do not always provide sufficient or clear information, which can limit patients’ understanding and compromise truly informed consent, as required by ethical guidelines for research involving human participants. As a result, some patients may take part in studies without fully understanding the purpose of the research, potentially affecting the relevance and quality of their responses. Participants therefore suggested that involving patients in the review and development of participant information sheets, informed consent forms, and questionnaires could help address these challenges. For example, a participant identified a potential role in

Preparing study documents for the participants (Cancer and diabetic patient, Ghana, P007).

They also highlighted a desire to feel recognised and to understand how their contributions benefit the wider community. Meaningful engagement throughout the research process, alongside capacity building to support effective communication with researchers, was viewed as central to ensuring that patient involvement is both ethical and impactful.

Participants also recognised that contributing to the development and use of questionnaires would require appropriate preparation and training.

Communication like how to communicate the research findings to the whole public and also it depends on the research topics so how to go about with the questionnaires, and I know when that time comes you would be trained for it (Diabetes and hypertension patient, Ghana, P001).

##### Translation of study tools

Given the cultural and linguistic diversity within each country, participants emphasized the importance of providing information in languages they are familiar with. They noted that community members could actively participate in translating study materials into local languages, such as Chewa and Yao in Malawi, Luganda and Swahili in Uganda, Shona and Ndebele in Zimbabwe, and Ewe and Nzema in Ghana. Participants highlighted that many researchers do not speak minority languages, which can limit effective communication. Although each country has a dominant language, elderly individuals often feel more respected and included when addressed in a language they know. For instance, Yao a minority language in Malawi is widely spoken by older adults in the areas where data were collected, and using it fosters better engagement.

….I can help with translation from Chewa to Yao, I was able to translate to the doctor in the hospital (Caregiver, Malawi, C004).

##### Recruitment of study participants and new representatives

Recruitment of participants was identified as a critical aspect of any research study. Many potential participants spend most of their time at home due to illness, making it difficult for them to be aware of ongoing research activities at their facilities unless they visit the hospital for clinical appointments. Participants suggested that engaging patients and their families who have similar conditions or needs could help increase awareness about research studies. They also noted that within their communities, there are existing structures and leadership, such as religious leaders, traditional chiefs, or village leaders, some of whom themselves have long-term conditions. These structures can be leveraged to identify and reach potential participants more effectively. Additionally, participants observed that messages about research are often clearer and more persuasive when communicated by someone with similar experiences.

Meaningful patient engagement was further emphasized as essential for strengthening participation in research. Establishing patient engagement forums should place patients at the center of the process, allowing them to lead efforts to involve others. Participants highlighted that when information comes from a fellow patient rather than a researcher, people are more likely to trust it, as they are often skeptical about potential negative impacts on their treatment.

IThe best way could be, giving them enough awareness messages about the research so that they can understand what it means, this can help to change their mind due to the way how you have given them the message (Caregiver, Ghana, C006).

I would involve chiefs so that they can convince those who don’t understand. I can convince the chief about the goodness of taking part in research (Cancer and hypertension patient, Malawi, P005).

##### Data collection

Participants reported that they sometimes have difficulty understanding the questions posed by researchers during studies. This is often due to the absence of a shared language or because the questions are too technical for them to fully comprehend. They felt that, since they can relate to and understand these questions, they are well-positioned to ask them on behalf of other patients in similar situations.

Participants also emphasized the need for researchers to be flexible regarding the timing and location of data collection. Some patients, particularly the elderly or those who are very ill, may be unable to travel to the hospital, making home-based data collection necessary. Additionally, some potential participants live in hard-to-reach areas. Involving local participants as data collectors can help ensure that the perspectives and experiences of these individuals are included when designing interventions that affect them.

You have to visit them at home, because some of the elderly people cannot walk, they are sick while they are at home, and it is even difficult to carry them to the hospital. Some places are just too difficult for others to reach, but for us we have no choice we live there.

(Caregiver, Malawi, C005).

Another caregiver highlighted that home-based involvement was particularly important for frail older adults who were unable to travel to health facilities:

It’s good for elderly people to take part in research because sometimes there might not be young people to take them to the hospital so those conducting research should come and help them while still at home. **(**Caregiver, Malawi, C014**).**

##### Data analysis

A few participants reported that they want to be involved with data analysis. Most of them stated that they have not been involved in this process before, therefore they will find in difficult to engage through data analysis.

.… You have gone to various places and collected them, how you come about combining them together. You are analysing everybody’s answer. That knowledge I like. (Caregiver, Ghana 003).

##### Dissemination of study findings

Participants highlighted the importance of sharing study findings or investigation results with the communities involved. There was a general perception that researchers often engage participants during the study, but rarely return to communicate the outcomes once the study is completed. Participants expressed a strong desire to be involved in disseminating this information to their colleagues and communities, leveraging existing community structures such as religious institutions, traditional leaders or chiefs, and local radio and television stations.

They also suggested that leaflets or information sheets summarizing research findings could be prepared, and indicated their willingness to participate in developing these materials in context-specific languages and distributing them to relevant stakeholders within their communities.

The best way is to publicise through radios, televisions or gathering them together and inform them what was done and the results (Caregiver, Zimbabwe C012).

#### Patient engagement forums

##### Consultation fostered through structured patient engagement platforms

Participants emphasized the need for structured patient engagement platforms, such as patient forums or advisory councils, to enable meaningful interaction with researchers, policymakers, and healthcare providers. They proposed that these platforms should meet regularly and that patients should be empowered with the skills and support needed to participate effectively.

Have a patient group on research which can meet say every month where patients can avail their information (Focus group discussion, Men’s group, Uganda).

Need a patient platform such as this one for sharing and prior communication to enable participation (Focus group discussion, Men’s group, Uganda).

Participants viewed patient engagement as central to the development of truly patient-centred palliative care services. Importantly, participants expressed a strong desire to be involved not only in identifying challenges related to their care but also in co-developing solutions that extend beyond clinical treatment alone, but also in contributing to decisions about research and service delivery.

Patients should be involved and their input should be considered over and beyond the opinions of doctors and similar ranked persons (Focus group discussion, Women’s group, Uganda).

Participants further highlighted the need for deeper and more sustained engagement with researchers beyond being recruited as study participants. They expressed interest in contributing to the dissemination of research findings, participation in ethics committees, and involvement in setting priority research agendas. The importance of feedback was illustrated by one participant’s experience:

Many times researchers come and take information including samples but they never come back to share results of this information (Focus group discussion, Women’s group, Uganda).

##### Structure of patient forums

Participants across the four countries, and within individual country contexts, proposed varying frequencies for patient engagement meetings. While participants expressed a strong desire for regular engagement, they were also cognisant of the financial and logistical implications of convening such meetings. As a result, they emphasized the importance of aligning meeting frequency with the availability of resources to ensure sustainability.

To balance efficiency and effectiveness, participants suggested several feasible options for engagement. Three main preferences emerged from the focus group discussions and interviews: monthly meetings, quarterly engagements, and biannual meetings. These variations reflect differing local contexts, resource constraints, and participant availability, underscoring the need for flexible, context-specific approaches to structuring patient and public involvement and engagement activities.

We can have a patient group on research which can meet every month where patients can share their information (HIV and cancer patient, Malawi, P008).

Participants further emphasized the importance of being informed well in advance about scheduled meetings to allow adequate planning. They also expressed a strong preference for meetings to be held close to their place of residence or at easily accessible venues. This was viewed as essential to minimise transport costs and physical burden, and to ensure timely access to healthcare facilities in the event that participants became unwell during or after meetings.

I think these meetings she be arranged just like you did, through sending messages by phone in time at least 5 days earlier, so that we will be aware because my daughter is the one with the phone number that receives these messages (Caregiver, Zimbabwe 008).

May be if I can fall sick suddenly, but if the venue can be near although I don’t have money for transport I can walk (Caregiver, Malawi, C015).

#### Sustaining PPIE activities

##### Availability of resources

While some patients can be easily reached within hospital premises, effective patient engagement and involvement require the allocation of adequate resources. Treatment for serious illnesses such as cancer, stroke, and heart failure is often complex and prolonged, with patients attending periodic appointments for ongoing assessment and management. These appointments are scheduled over extended periods, and depending on the condition, some patients attend monthly visits, incurring significant transport costs.

Participants noted that when patient engagement forums or research activities require the involvement of specific patients, careful planning and appropriate approaches are essential. Even with minimal resources, meaningful patient and public involvement and engagement (PPIE) activities can be successfully implemented. Participants also emphasized the importance of being acknowledged for their time spent in engagement activities and research participation. Although they recognized that their involvement is voluntary, they felt that providing a small token of appreciation would be a positive gesture. They suggested monetary support to help cover basic needs, such as food, which would further motivate proactive participation.

Across all four countries, participants consistently reported the need for a dedicated budget to support sustained patient engagement and involvement in service development, research, and ethics processes. They indicated that such a budget could be used to hire venues for meetings, provide transport reimbursements, and compensate participants for their time. Additionally, participants highlighted the importance of providing refreshments, such as water and snacks, during data collection activities, noting that many patients travel long distances to participate in research, often without having had breakfast.

Have some transport since some of us may not be able to afford to move. (Caregiver, Zimbabwe C006).

We can always have these meetings when resources allow (Caregiver, Malawi C012).

Maybe receiving some small cash as a token of appreciation for taking part in research (Caregiver, Malawi C002).

Participants also recognised that involvement offered important non-financial benefits, particularly opportunities to gain knowledge and increase confidence.

Besides knowledge, once one gains knowledge it means I am now more enlightened. **(**Caregiver, Zimbabwe, C010).

##### Training and capacity building

Participants expressed strong interest in developing the knowledge and skills required to effectively engage in palliative care research activities. They identified research training as a critical developmental need to enable them to lead such initiatives, including gaining a clear understanding of what research is, how it is conducted, and why it is undertaken. Participants also expressed interest in learning about specific research processes, including data analysis.

…. You have gone to various places and collected them, how you come about combining them together. You are analysing everybody’s answer. That knowledge I like. (Caregiver, Ghana, 003).

Participants further recognised that contributing to research activities would require appropriate preparation and training, particularly in relation to questionnaires and communicating research findings.

Communication like how to communicate the research findings to the whole public and also it depends on the research topics so how to go about with the questionnaires, and I know when that time comes you would be trained for it (Diabetes and hypertension patient, Ghana, P001).

These findings highlight the importance of providing accessible, role-specific training to enable patients and caregivers to contribute meaningfully to research activities.

Another key developmental need identified was training in data collection and management. Participants noted that while they valued the opportunity to engage other patients in discussions about their illnesses, they required adequate preparation to ensure accurate and meaningful data collection. They also highlighted the importance of training and ongoing support to build confidence in undertaking these tasks. Participants emphasized that to effectively lead all aspects of patient and public involvement and engagement (PPIE), regular guidance and follow-up from experienced mentors would be essential.

#### Barriers to the implementation of PPIE activities

##### Language and literacy diversity

All participating countries are characterized by significant ethnic and linguistic diversity, with multiple tribes and languages spoken within each country. As a result, streamlining communication to ensure that all patients are reached is an essential component of effective service delivery. Patients reported numerous challenges related to communication within this context. Addressing language barriers is therefore critical, even when multiple communication channels are used, and must be done while also considering factors such as varying levels of literacy among patients.

Barrier in communication from the gate to the doctor with limited effort to try and translate information to patients. Gatemen speak Luganda even when patients come from diverse backgrounds (Focus group discussion, Men’s group, Uganda).

What I will lack on translations is that I can’t manage English, but Chichewa and Chiyao, I can manage (Caregiver, Malawi, C002).

Had it been I know how to write I could contribute properly but since I do not know how to write so it’s difficult (Hypertension, diabetes and cancer patient, Malawi, P003).

##### Lack of clear information

Participants emphasized the need to be adequately informed about their involvement in palliative care research. They highlighted the importance of providing clear and detailed explanations of expectations, as some individuals may have high expectations of personal benefit from participation. Participants therefore stressed that, at the outset of a research project, potential members of the public should be clearly informed about their roles and responsibilities, as well as what they can realistically expect to gain from their involvement.

The barriers could be the benefits which people want to get, because some people get involved in a program in order to get something, but some are there for voluntary work so that they can learn and teach others about health issues, so for those who need benefits cannot be active. If they see that they are not getting anything they expected they can easily abandon the group (Caregiver, Malawi, C005).

The biggest problem is lack of understanding in the community, the problem is that when you are working with researchers, people think that you are earning a lot of money from the government even if you are earning a small amount (Diabetes and hypertension patient, Malawi, P013).

Participants therefore felt it is very important to manage expectations through communication and information provision at the onset of the research project

The best solution is that there needs to be a good communication between the researchers or from the government and the community because most of the times when you promise people from the village that you are coming and you didn’t keep your promise those people in village feel betrayed and you can’t get the information you were looking for (Hypertension, diabetes and arthritis patient, Zimbabwe, P004).

##### Financial barriers

Participants identified financial constraints as the most significant barrier to implementing patient and public involvement and engagement (PPIE) activities. They reported already incurring substantial costs related to healthcare, including transport expenses to access services, which made it difficult to travel solely to attend PPIE meetings. Additionally, participants noted that ongoing financial support is necessary for the effective functioning of PPIE groups, including covering costs associated with making phone calls to other members or visiting them at home to share information and provide reminders about upcoming PPIE meetings.

The barriers could be the issue of financial problem, may be some of the members will be coming from far areas which may require transport, then may be on the day of meeting it can be that the person has no money for transport this can be a barrier (Stroke, hypertension, and diabetes patient, Malawi P014).

…… a group cannot function without money, so the resources can be a major barrier, but if resources are there then other barriers will be reduced (Caregiver, Zimbabwe, C012).

## Discussion

In this study, patients and caregivers reported strong motivation to participate in palliative care research and to use patient engagement forums as platforms to voice concerns. Participants expressed a desire to be actively involved across multiple stages of the research process, including recruitment of study participants, provision of psychological support to fellow participants, development of study tools, data collection, data analysis and dissemination of research findings. The findings suggest that participants viewed involvement not only as an opportunity to contribute to research, but also as a means of having their experiences recognised and informing decisions about research and care. They emphasized the importance of leveraging existing community structures such as religious leaders and traditional authorities to support participant identification, recruitment, and dissemination of findings. Participants also highlighted the need to consider cultural practices and norms when designing and conducting research activities.

The use of community structures for disseminating research findings was strongly emphasized. In many African settings, these structures represent powerful and trusted platforms for information sharing, often reaching large audiences using locally available resources. For example, church gatherings and funeral ceremonies frequently bring together hundreds of community members and are typically led by religious or traditional leaders. Participants’ suggestions to engage religious and traditional leaders, alongside local radio and television, indicate the potential value of using familiar communication channels to make research findings more accessible to communities. Engaging these structures has the potential to increase the reach and impact of research findings. Previous studies in Kenya and Ghana have similarly reported positive outcomes associated with community engagement in research dissemination [18, 19].

However, the acceptability and effectiveness of different dissemination approaches should be assessed within the communities in which they are implemented. This study provides participant-informed recommendations for developing PPIE approaches for people living with serious illness in sub-Saharan Africa. Rather than demonstrating that PPIE structures have already been established or are effective, the findings identify practical considerations that can inform their development, implementation, and evaluation.

Our findings align with previous research conducted in the United Kingdom, where we demonstrated that building and sustaining relationships, adopting flexible approaches, and identifying the “right” people were key to successful public involvement. Strong relationships create safe spaces for discussing sensitive issues, which are common in palliative care research [20].

We engaged a diverse group of patients with a range of chronic and life-limiting conditions including cancer, HIV, diabetes, chronic obstructive pulmonary disease (COPD), and heart failure reflecting populations that frequently access primary and chronic care services, particularly older adults with multimorbidity. The inclusion of patients and caregivers from four countries provided opportunities to explore perspectives across different health-system, cultural, and linguistic contexts. The findings are consistent with person-centred principles that recognise patients and caregivers as partners whose experiences and priorities should inform research decisions [21]. Establishing trust, providing clear information, and creating opportunities for meaningful contribution emerged as important considerations for future PPIE implementation.

Sustainability of PPIE activities was emphasized by participants across all four countries. Ensuring stakeholder diversity and inclusiveness has been identified as a critical component of sustainable PPIE [22]. Participants highlighted the importance of well-established group structures, clear leadership, regular meetings, and adequate financial resources to maintain engagement. Recognition of participants’ time and contributions both as research participants and patient representatives was also seen as essential. This work further demonstrate that participants wanted regular opportunities to share information, contribute to decisions, and receive feedback on research findings. These findings suggest that structured forums could provide a mechanism for moving beyond one-off consultation towards more sustained relationships between researchers and communities.

Harrison et al.‘s narrative review, drawing predominantly on evidence from North America and Europe, identified key principles for sustainable patient engagement in research, including respect, equitable partnerships, trust, training, financial support, and bidirectional communication [22]. Our findings are consistent with these principles and identify context-specific considerations for their application in palliative care research in sub-Saharan Africa. Participants generated context-specific recommendations, including engaging traditional and religious leaders, translating materials into local languages, conducting home-based engagement for seriously ill patients, establishing patient engagement forums, and adopting flexible approaches that accommodate resource constraints and multimorbidity. These recommendations provide a foundation for developing and testing PPIE approaches in resource-limited settings, rather than evidence that such approaches have already achieved sustained engagement or improved research outcomes. PPIE in health research requires substantial investment of time, financial resources, and emotional and intellectual effort from research institutions, patients, and the wider public [23].

The findings also have implications for the application of person-centred principles to research partnerships. Participants’ emphasis on representation, meaningful input into decisions, and opportunities to contribute across the research process reflects the importance of recognising lived experience as a source of knowledge. The study therefore offers a contextually relevant foundation for developing PPIE structures and networks, while further implementation research is required to establish their feasibility, acceptability, sustainability, and impact.

Participants also expressed a strong need for training to effectively engage in PPIE activities. Priority areas included understanding the research process, research methods, and dissemination strategies for diverse audiences. Our data demonstrate participants’ interest in learning about data analysis and their recognition that contributing to questionnaires and communicating findings would require appropriate preparation. These findings support the provision of accessible, role-specific training tailored to participants’ existing knowledge, experience, and preferred research roles. PPIE requires a broad skill set, including interpersonal, administrative, and coordination skills. Previous studies have shown that collaborative research environments depend on trust, respect, honesty, and effective communication [22, 24, 25]. Participants were particularly interested in contributing to recruitment, data collection, tool development, translation of materials into culturally appropriate languages, and data analysis findings consistent with earlier research on patient preferences for involvement in research [22].

Although the present findings primarily support the need for training and capacity building, ongoing mentorship and supportive supervision may provide additional mechanisms for consolidating skills and building confidence in PPIE roles. These approaches should be explored and evaluated in future implementation work rather than presented as established requirements arising directly from the participant data.

Participants also described insufficient information about research and uncertainty regarding the roles and expectations associated with involvement. These findings highlight the importance of clear communication, transparent expectations, and accessible information throughout the research process. Previous research has similarly identified challenges faced by researchers, including difficulty defining roles, managing expectations, and providing appropriate educational materials about research processes [26–28]. Future PPIE initiatives should therefore consider how researchers and patient representatives can be supported to establish shared expectations, clarify responsibilities, and maintain bidirectional communication.

### Strengths and limitations

This study explored patient and caregiver perspectives on meaningful involvement in palliative care research across four sub-Saharan African countries.

Participants were recruited from four countries and included both patients and caregivers with diverse conditions. A purposive sampling framework was employed across all settings, resulting in a diverse sample in terms of culture, language, tribe, and disease condition. Notably, differences across countries were not prominent in the study findings.

Research rigor was strengthened through the involvement of local researchers in data collection, transcription, translation, and analysis. A coding framework was collaboratively developed and agreed upon by the research team.

Some limitations should be acknowledged. First, the study populations and methods differed across countries. In Uganda, data were collected through focus group discussions with adult cancer patients, whereas individual interviews were conducted with older adults living with multimorbidity and their caregivers in Ghana, Malawi, and Zimbabwe. No caregivers were recruited in Uganda. These differences may have influenced the perspectives expressed and limit direct comparisons between countries and participant groups.

In Ghana and Malawi, senior local researchers did not speak all local languages and therefore relied on independent language experts for translation. Although interviewers were consulted to preserve the intended meaning, some linguistic and cultural nuances may have been lost during transcription and translation.

Finally, this was an exploratory qualitative study of participants’ perspectives and preferences. It did not implement or evaluate the proposed PPIE structures, training approaches, or mentorship arrangements. Consequently, the findings cannot establish their effectiveness, sustainability, or impact on research quality, healthcare delivery, or patient outcomes. Further research is needed to co-develop, implement, and evaluate these approaches in different settings, including assessment of the resources and support required for sustained involvement.

## Conclusion

Patient and public involvement and engagement (PPIE) in research provides an important opportunity to co-develop relevant research questions and to design, implement, and disseminate people- and community-centred research. Palliative care research often addresses emotionally sensitive topics and involves diverse populations living in complex and unpredictable circumstances.

This study provides participant-informed recommendations for developing meaningful and sustainable PPIE in palliative care research in sub-Saharan Africa. These include establishing structured patient engagement platforms, involving patients and caregivers across the research process, providing appropriate training and mentorship, and ensuring adequate resources and flexible approaches that respond to local contexts and individual needs.

Meaningful engagement and involvement in palliative care research should be grounded in respectful partnerships and recognise patients and caregivers as active contributors to research. This study highlights the need for strategic approaches to public involvement within palliative care research institutions to support the sustainability of PPIE initiatives. The findings offer a contextually relevant foundation for the development of PPIE structures and networks within palliative care research institutions. Future research is needed to explore how these approaches can be effectively refined, implemented and evaluated to identify the local resources required to support and sustain PPIE in sub-Saharan Africa.

## Data Availability

The datasets generated and/or analysed during the current study are not publicly available because they contain potentially identifiable qualitative interview and focus group data from participants, and public sharing could compromise participant confidentiality and the conditions under which ethical approval and informed consent were obtained. The datasets are, however, available from the corresponding author on reasonable request, subject to institutional ethical approval and any applicable data-sharing agreements.

## Abbreviations

COPD: Chronic obstructive pulmonary disease
FGDs: Focus group discussions
GHS: Ghana health service
HIV: Human immunodeficiency virus
LMICs: Low- and middle-income countries
MRCZ: Medical research council of Zimbabwe
NCDs: Non-communicable diseases
PPI: Patient and public involvement
PPIE: Patient and public involvement and engagement
SD: Standard deviation
UNCST: Uganda national council for science and technology
WHO: World health organization
